# Correction to ‘Ablation of PRMT6 reveals a role as a negative transcriptional regulator of the p53 tumor suppressor’

**DOI:** 10.1093/nar/gkag109

**Published:** 2026-02-03

This is a correction to: Mathieu Neault, Frédérick A. Mallette, Gillian Vogel, Jonathan Michaud-Levesque, Stéphane Richard, Ablation of PRMT6 reveals a role as a negative transcriptional regulator of the p53 tumor suppressor, Nucleic Acids Research, Volume 40, Issue 19, 1 October 2012, Pages 9513–9521, https://doi.org/10.1093/nar/gks764

In September 2025, concerns about overlap between two panels in Figure 4 were raised via PubPeer (https://pubpeer.com/publications/70B54DEAFAF881D442DA2CF96BD08C#1). The authors now wish to correct Figure 4.

According to the authors, the images in Figure 4 were accidently duplicated due to a labelling error.

The authors provided original or duplicate data supporting this figure to the journal editors, and it is available in the Supplementary Information to this correction notice.

This correction does not affect the results, discussion and conclusions presented in the article. These details have been corrected only in this correction notice to preserve the published version of record.


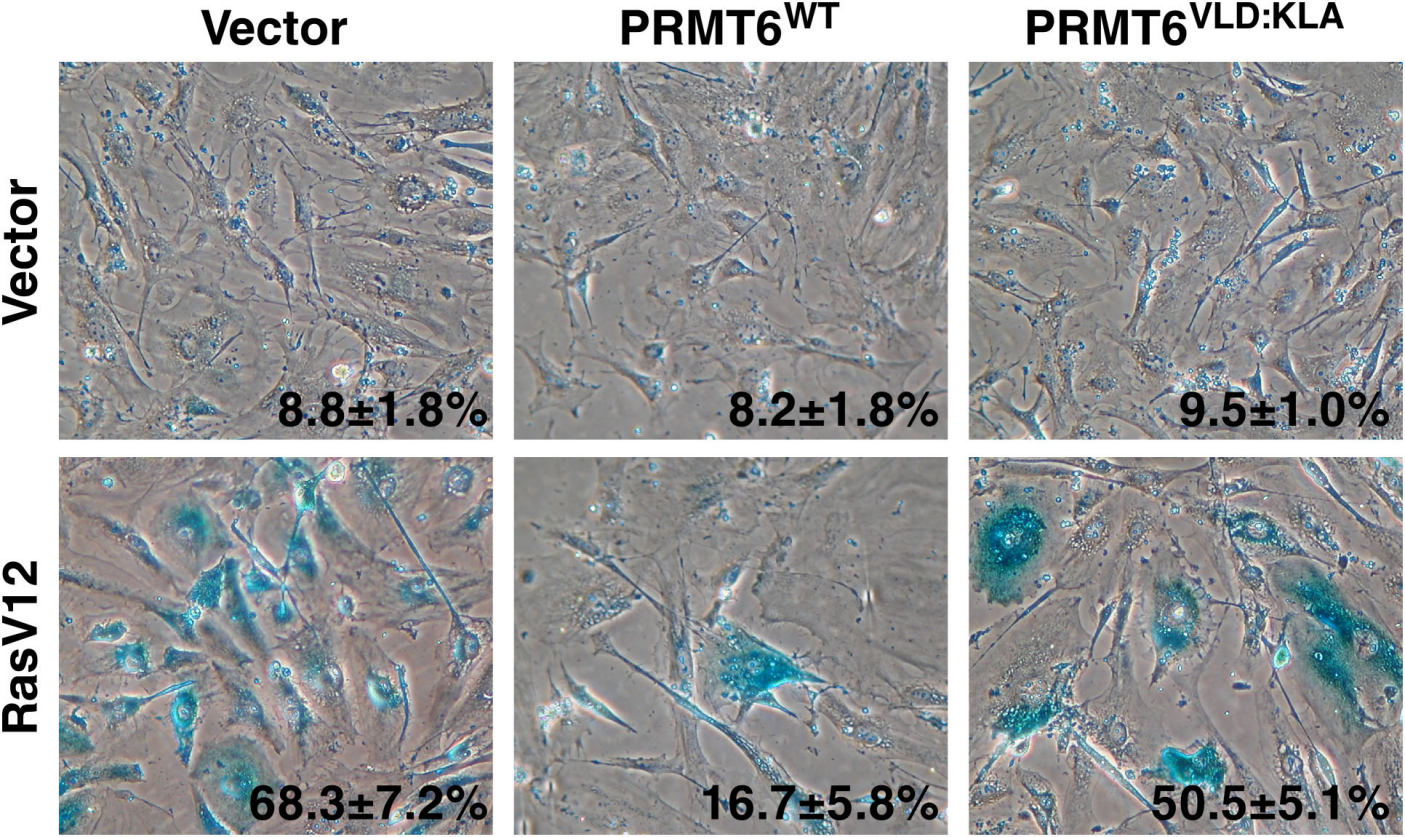


Corrected Figure 4

## Supplementary Material

gkag109_Supplemental_Files

