# Supplementary figures and images for: Correction to ‘Ablation of PRMT6 reveals a role as a negative transcriptional regulator of the p53 tumor suppressor’
Source: Nucleic Acids Res. 2026 Feb 3;54(3):gkag109. doi: 10.1093/nar/gkag109 (PMC12865448; doi:10.1093/nar/gkag109)

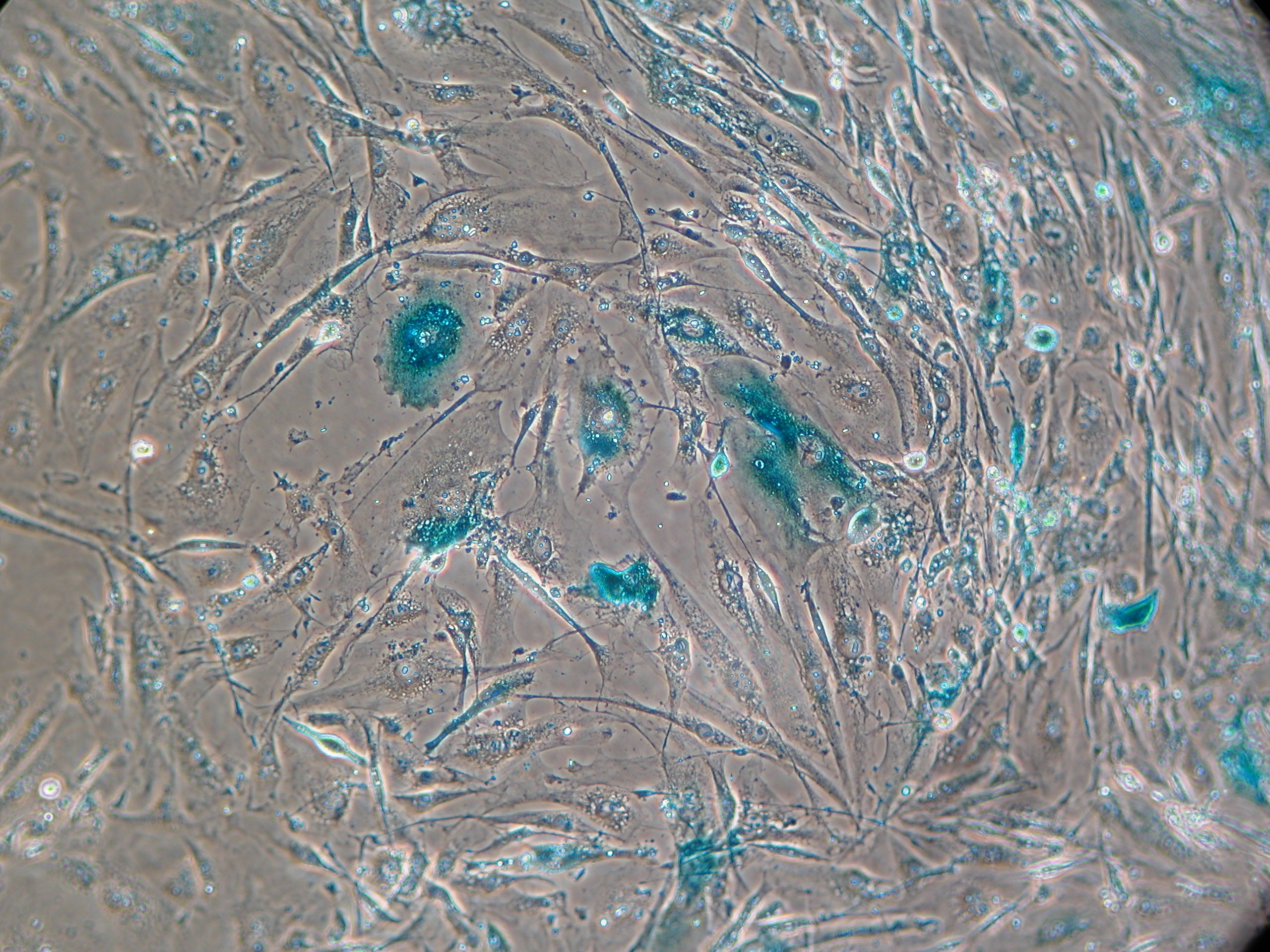

Supplement: gkag109_Supplemental_Files [file gkag109_supplemental_files.zip › Ras+PRMT6mut.JPG]

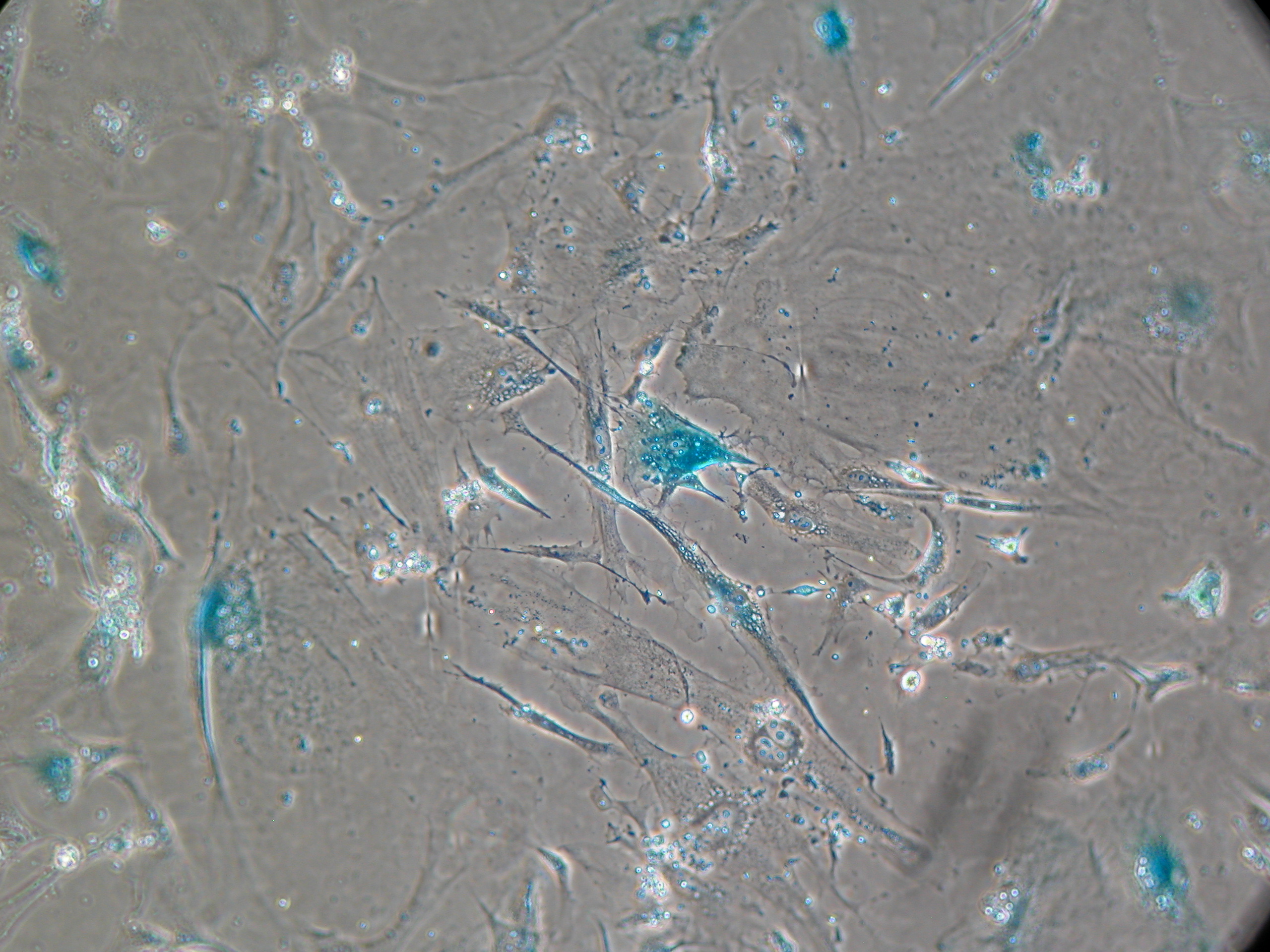

Supplement: gkag109_Supplemental_Files [file gkag109_supplemental_files.zip › Ras+PRMT6wt.JPG]

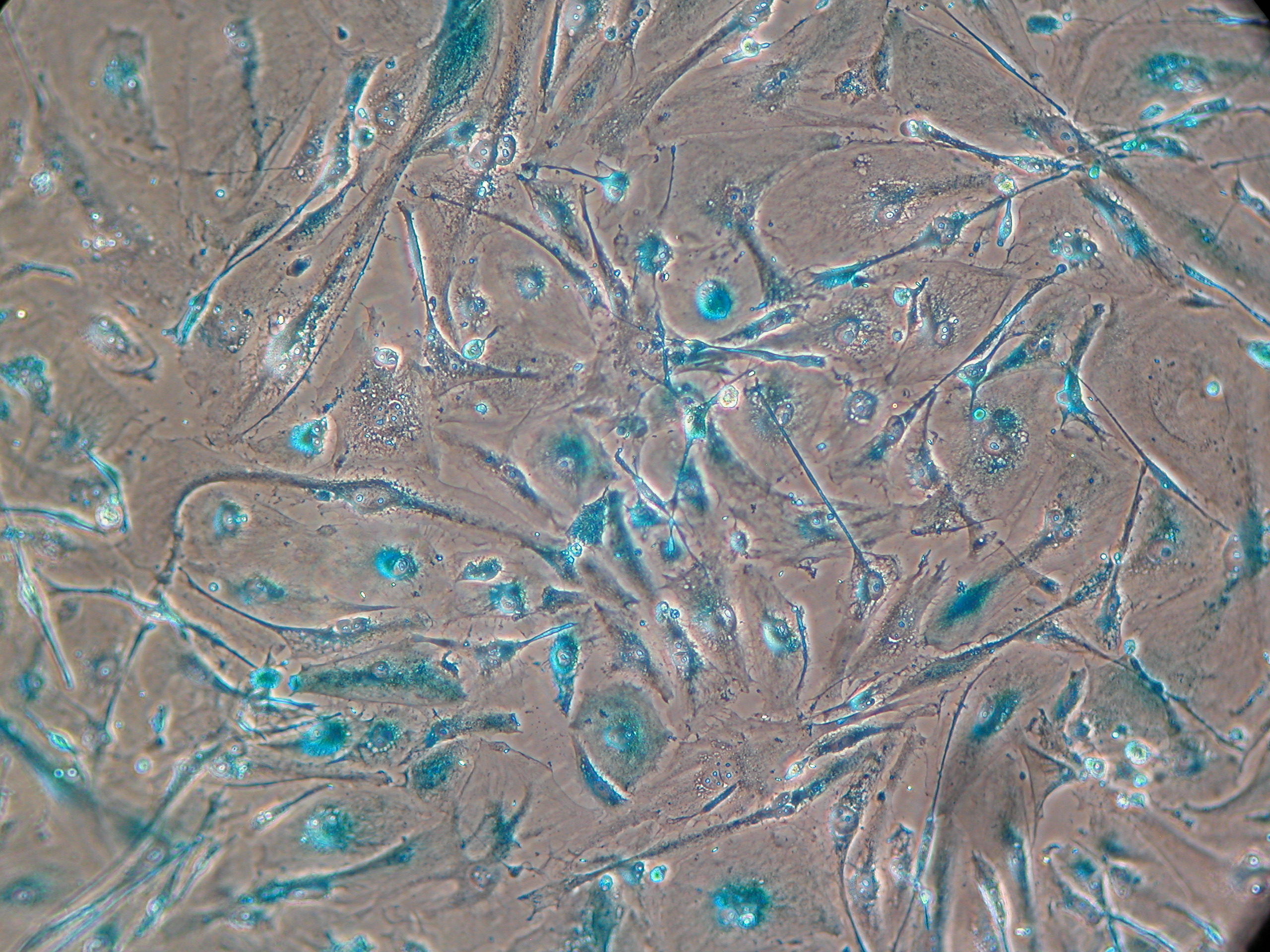

Supplement: gkag109_Supplemental_Files [file gkag109_supplemental_files.zip › Ras+Vector.JPG]

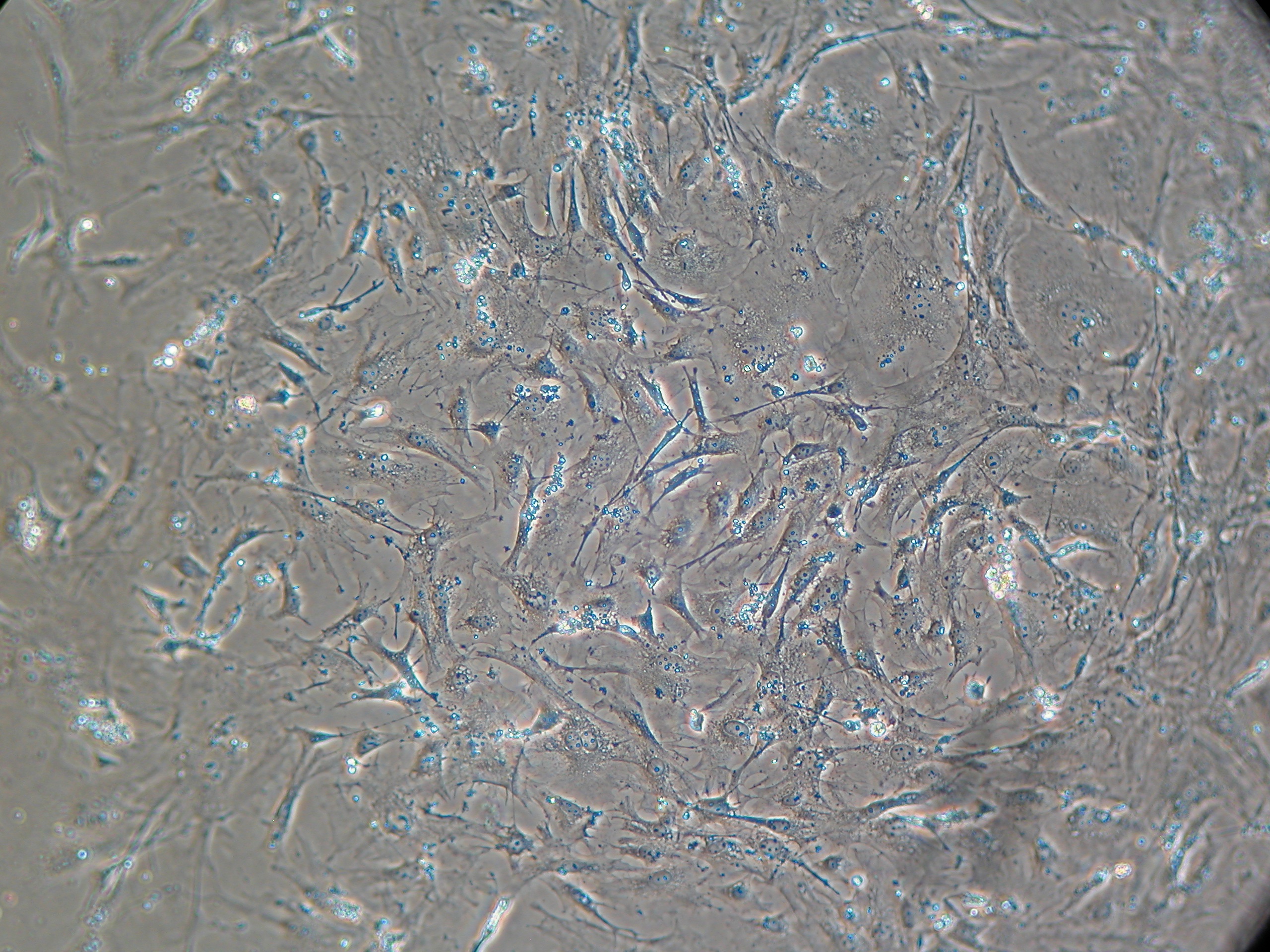

Supplement: gkag109_Supplemental_Files [file gkag109_supplemental_files.zip › Vector+PRMT6mut.JPG]

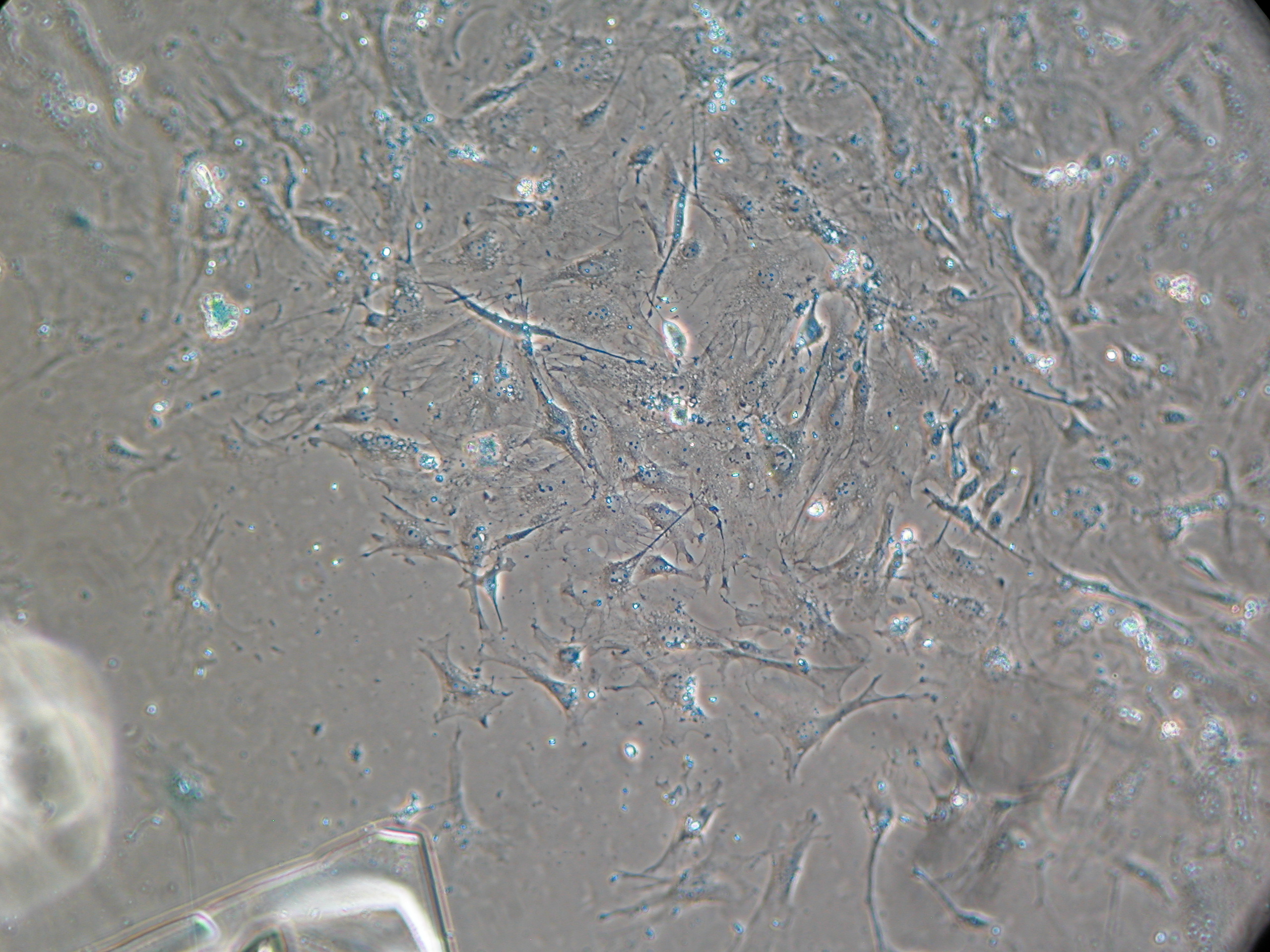

Supplement: gkag109_Supplemental_Files [file gkag109_supplemental_files.zip › Vector+PRMT6wt.JPG]

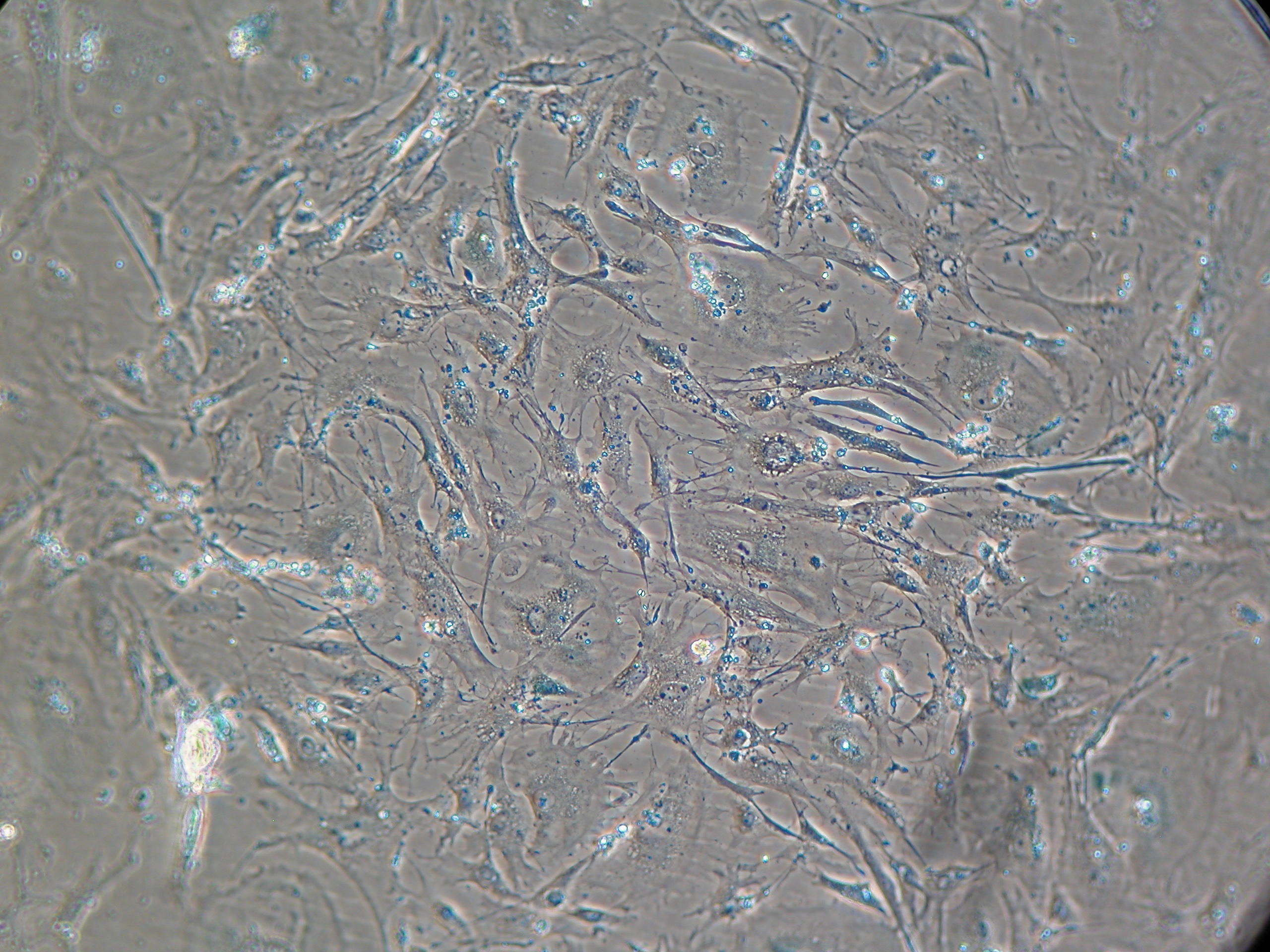

Supplement: gkag109_Supplemental_Files [file gkag109_supplemental_files.zip › Vector+Vector.JPG]
